# The genome sequence of the Grey Arches moth, *Polia nebulosa *Hufnagel, 1766

**DOI:** 10.12688/wellcomeopenres.23608.1

**Published:** 2025-02-03

**Authors:** Andy Griffiths, Tom Prescott, Ronald Forrester, Wellcome Sanger Institute Tree of Life Management, Samples and Laboratory team, Wellcome Sanger Institute Scientific Operations: Sequencing Operations, Wellcome Sanger Institute Tree of Life Core Informatics team, Tree of Life Core Informatics collective, Darwin Tree of Life Consortium

**Affiliations:** 1 Royal Botanic Garden Edinburgh, Edinburgh, Scotland, UK; 2 Tree of Life, Wellcome Sanger Institute, Hinxton, England, UK; 3 Butterfly Conservation Scotland, Stirling, Scotland, UK; 4 Independent researcher, Rothesay, Isle of Bute, Scotland, UK

**Keywords:** Polia nebulosa, Grey Arches moth, genome sequence, chromosomal, Lepidoptera

## Abstract

We present a genome assembly from an individual male specimen of *Polia nebulosa* (Grey Arches; Arthropoda; Insecta; Lepidoptera; Noctuidae). The genome sequence has a total length of 1,011.20 megabases. Most of the primary assembly (99.27%) is scaffolded into 30 chromosomal pseudomolecules, including the Z sex chromosome. The mitochondrial genome has also been assembled and is 15.39 kilobases in length.

## Species taxonomy

Eukaryota; Opisthokonta; Metazoa; Eumetazoa; Bilateria; Protostomia; Ecdysozoa; Panarthropoda; Arthropoda; Mandibulata; Pancrustacea; Hexapoda; Insecta; Dicondylia; Pterygota; Neoptera; Endopterygota; Amphiesmenoptera; Lepidoptera; Glossata; Neolepidoptera; Heteroneura; Ditrysia; Obtectomera; Noctuoidea; Noctuidae; Hadeninae; *Polia*; *Polia nebulosa* Hufnagel, 1766 (NCBI:txid988024)

## Background

*Polia nebulosa*, commonly known as the Grey Arches, is a moth in the family Noctuidae, first described by Johann Siegfried Hufnagel in 1766. It is distributed across temperate Europe and Asia, extending as far east as Japan, but is absent from northernmost Fennoscandia and the southern parts of the Iberian Peninsula, Italy, and Greece. In mountainous regions such as the Alps, it can be found at elevations up to about 1,600 metres. It is fairly widespread throughout England, Scotland and Northern Ireland ( Kimber, 2024).

Adult wingspans range from 41–55 mm, and the forewing coloration is highly variable, from creamy white to dark brown or blackish. This species chiefly inhabits woodland areas; its larvae feed on low herbaceous plants in autumn and then switch to the buds and leaves of trees such as birch ( *Betula)* and willow ( *Salix* spp.) after hibernation ( NBN Atlas Partnership, 2024). The adults, which fly in June and July, are attracted to both light and sugar ( NBN Atlas Partnership, 2024).

**Figure 1.**
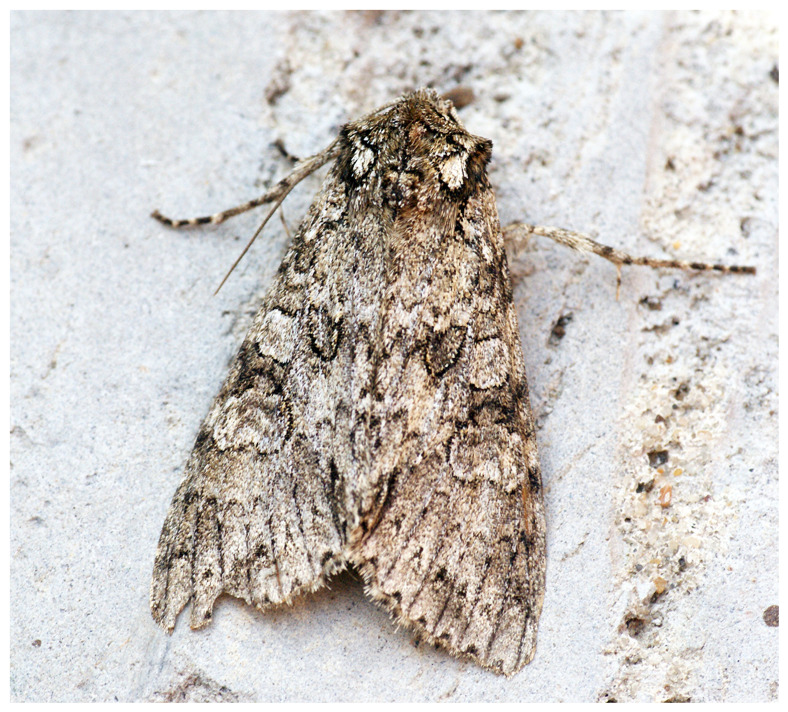
Photograph of *Polia nebulosa* by *Ben Sale* (not the specimen used for genome sequencing).

The genome of the Grey Arches, *Polia nebulosa*, was sequenced as part of the Darwin Tree of Life Project, a collaborative effort to sequence all named eukaryotic species in the Atlantic Archipelago of Britain and Ireland. Here we present a chromosomally complete genome sequence for *Polia nebulosa*, based on a male specimen from Glen Strathfarrar, Highlands, Scotland, United Kingdom ( Figure 1).

## Genome sequence report

The genome of *Polia nebulosa* ( Figure 1) was sequenced using Pacific Biosciences single-molecule HiFi long reads, generating a total of 25.49 Gb (gigabases) from 2.30 million reads, providing an estimated 24-fold coverage. Primary assembly contigs were scaffolded with chromosome conformation Hi-C data, which produced 128.16 Gb from 848.77 million reads. RNA data were also deposited. Specimen and sequencing details are summarised in Table 1.

**Table 1.** Specimen and sequencing data for *Polia nebulosa*.

Project information			
**Study title**	Polia nebulosa (grey arches)		
**Umbrella BioProject**	PRJEB61359		
**Species**	*Polia nebulosa*		
**BioSample**	SAMEA112198544		
**NCBI taxonomy ID**	988024		
Specimen information			
**Technology**	**ToLID**	**BioSample** **accession**	**Organism part**
**PacBio long read sequencing**	ilPolNebu2	SAMEA112198603	thorax
**Hi-C sequencing**	ilPolNebu1	SAMEA112198516	head
**RNA sequencing**	ilPolNebu2	SAMEA112198603	thorax
Sequencing information			
**Platform**	**Run accession**	**Read count**	**Base count (Gb)**
**Hi-C Illumina NovaSeq 6000**	ERR11242563	8.49e+08	128.16
**PacBio Sequel IIe**	ERR11242139	2.30e+06	25.49
**RNA Illumina NovaSeq 6000**	ERR11837483	6.97e+07	10.53
**RNA Illumina NovaSeq 6000**	ERR12035188	6.69e+07	10.1

Assembly errors were corrected by manual curation, including 55 missing joins or mis-joins and 15 haplotypic duplications. This reduced the assembly length by 0.55% and the scaffold number by 5.74%. The final assembly has a total length of 1,011.20 Mb in 114 sequence scaffolds, with 194 gaps, and a scaffold N50 of 33.7 Mb ( Table 2).

**Table 2.** Genome assembly data for *Polia nebulosa*, ilPolNebu2.1.

Genome assembly		
Assembly name	ilPolNebu2.1	
Assembly accession	GCA_951329385.1	
*Accession of alternate haplotype*	*GCA_951329505.1*	
Span (Mb)	1,011.20	
Number of contigs	309	
Number of scaffolds	114	
Longest scaffold (Mb)	80.95	
Assembly metrics *		*Benchmark*
Contig N50 length (Mb)	7.7	*≥ 1 Mb*
Scaffold N50 length (Mb)	33.7	*= chromosome N50*
Consensus quality (QV)	63.7	*≥ 40*
*k*-mer completeness	Primary: 79.17%; alternate: 76.79%; combined: 98.81%	*≥ 95%*
BUSCO v5.4.3 lineage: lepidoptera_odb10	C:98.9%[S:98.0%,D:0.9%], F:0.3%,M:0.8%,n:5,286	*S > 90%, D < 5%*
Percentage of assembly mapped to chromosomes	99.27%	*≥ 90%*
Sex chromosomes	Z	*localised homologous pairs*
Organelles	Mitochondrial genome: 15.39 kb	*complete single alleles*

* Assembly metric benchmarks are adapted from Rhie *et al.* (2021) and the Earth BioGenome Project Report on Assembly Standards September 2024. ** BUSCO scores based on the lepidoptera_odb10 BUSCO set using version 5.4.3. C = complete [S = single copy, D = duplicated], F = fragmented, M = missing, n = number of orthologues in comparison. A full set of BUSCO scores is available at https://blobtoolkit.genomehubs.org/view/ilPolNebu2_1/dataset/ilPolNebu2_1/busco.

**Figure 2.**
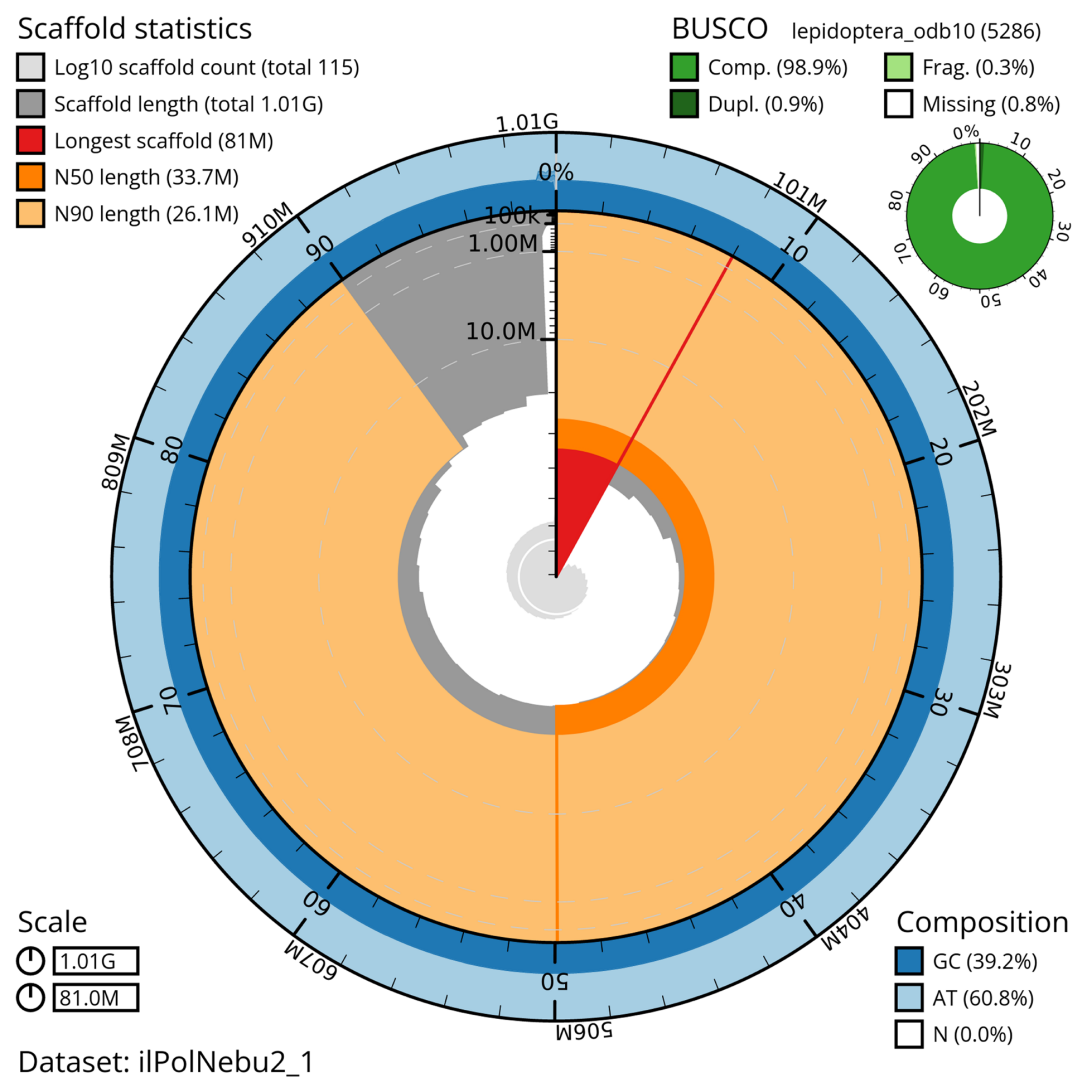
Genome assembly of *Polia nebulosa*, ilPolNebu2.1: metrics. The BlobToolKit snail plot provides an overview of assembly metrics and BUSCO gene completeness. The circumference represents the length of the whole genome sequence, and the main plot is divided into 1,000 bins around the circumference. The outermost blue tracks display the distribution of GC, AT, and N percentages across the bins. Scaffolds are arranged clockwise from longest to shortest and are depicted in dark grey. The longest scaffold is indicated by the red arc, and the deeper orange and pale orange arcs represent the N50 and N90 lengths. A light grey spiral at the centre shows the cumulative scaffold count on a logarithmic scale. A summary of complete, fragmented, duplicated, and missing BUSCO genes in the lepidoptera_odb10 set is presented at the top right. An interactive version of this figure is available at https://blobtoolkit.genomehubs.org/view/ilPolNebu2_1/dataset/ilPolNebu2_1/snail.

**Figure 3.**
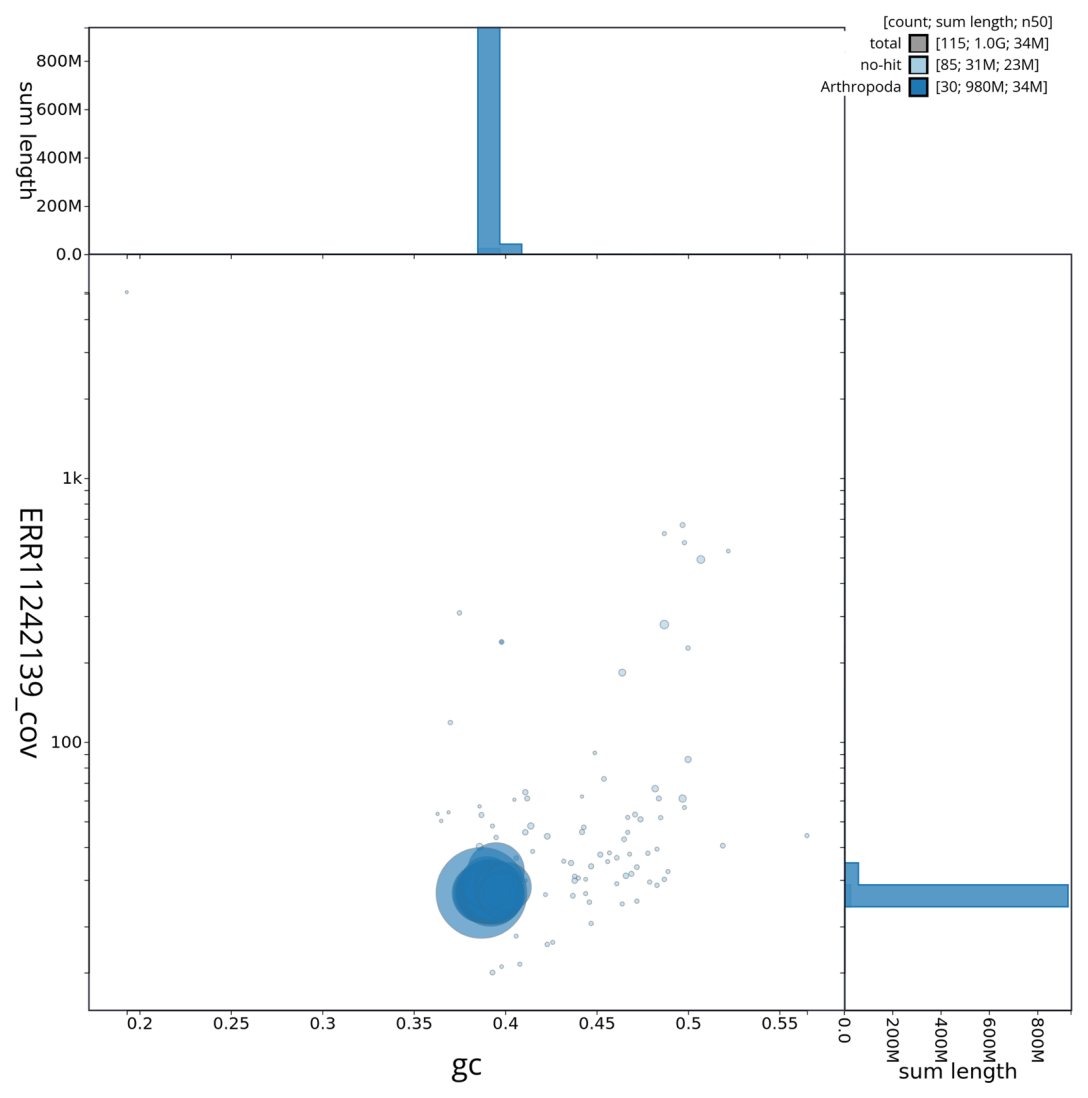
Genome assembly of *Polia nebulosa*, ilPolNebu2.1: BlobToolKit GC-coverage plot showing sequence coverage (vertical axis) and GC content (horizontal axis). The circles represent scaffolds, with the size proportional to scaffold length and the colour representing phylum membership. The histograms along the axes display the total length of sequences distributed across different levels of coverage and GC content. An interactive version of this figure is available at https://blobtoolkit.genomehubs.org/view/ilPolNebu2_1/dataset/ilPolNebu2_1/blob.

**Figure 4.**
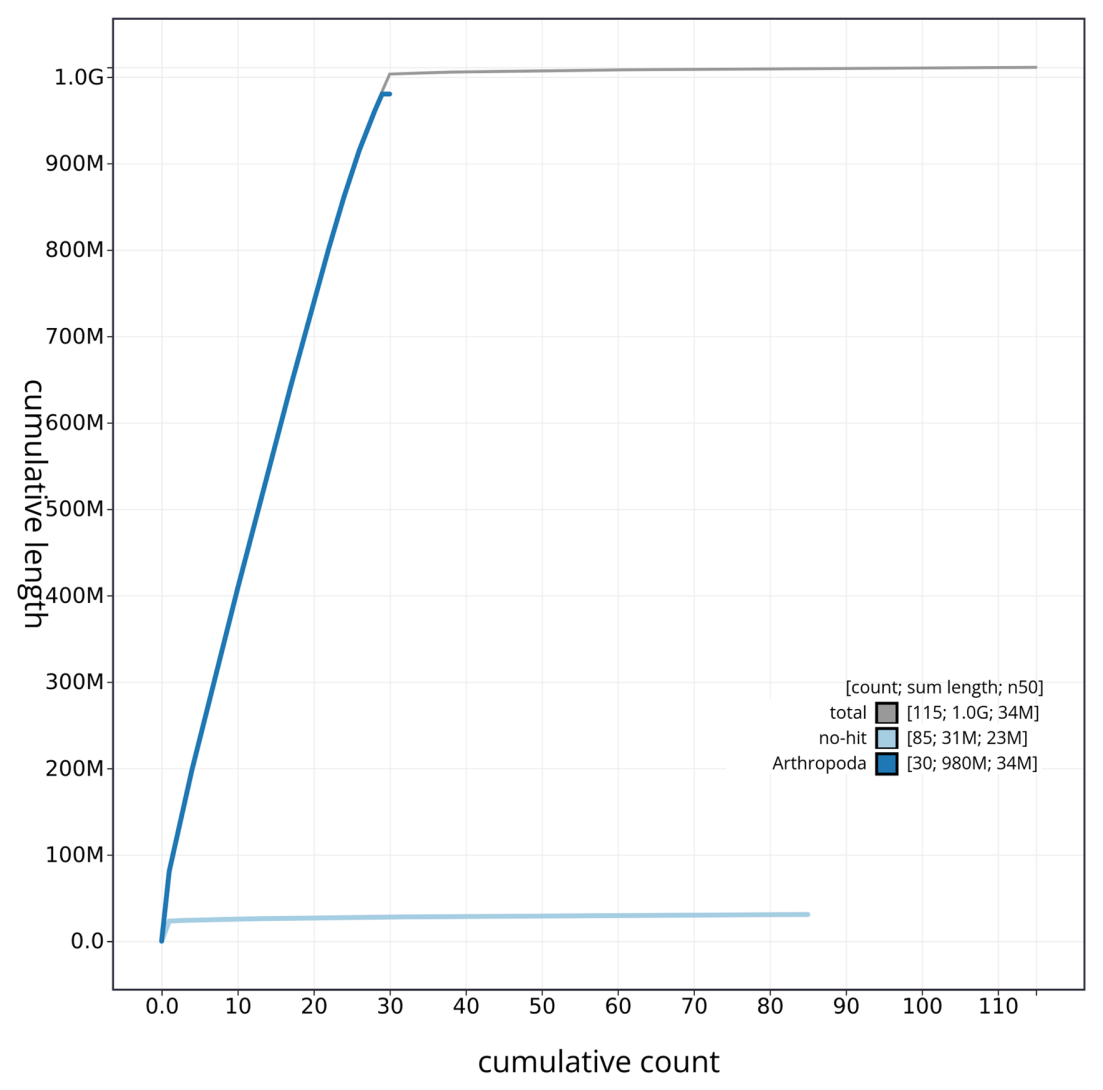
Genome assembly of *Polia nebulosa* ilPolNebu2.1: BlobToolKit cumulative sequence plot. The grey line shows cumulative length for all sequences. Coloured lines show cumulative lengths of sequences assigned to each phylum using the buscogenes taxrule. An interactive version of this figure is available at https://blobtoolkit.genomehubs.org/view/ilPolNebu2_1/dataset/ilPolNebu2_1/cumulative.

The snail plot in Figure 2 provides a summary of the assembly statistics, indicating the distribution of scaffold lengths and other assembly metrics. Figure 3 shows the distribution of scaffolds by GC proportion and coverage. Figure 4 presents a cumulative assembly plot, with separate curves representing different scaffold subsets assigned to various phyla, illustrating the completeness of the assembly.

**Figure 5.**
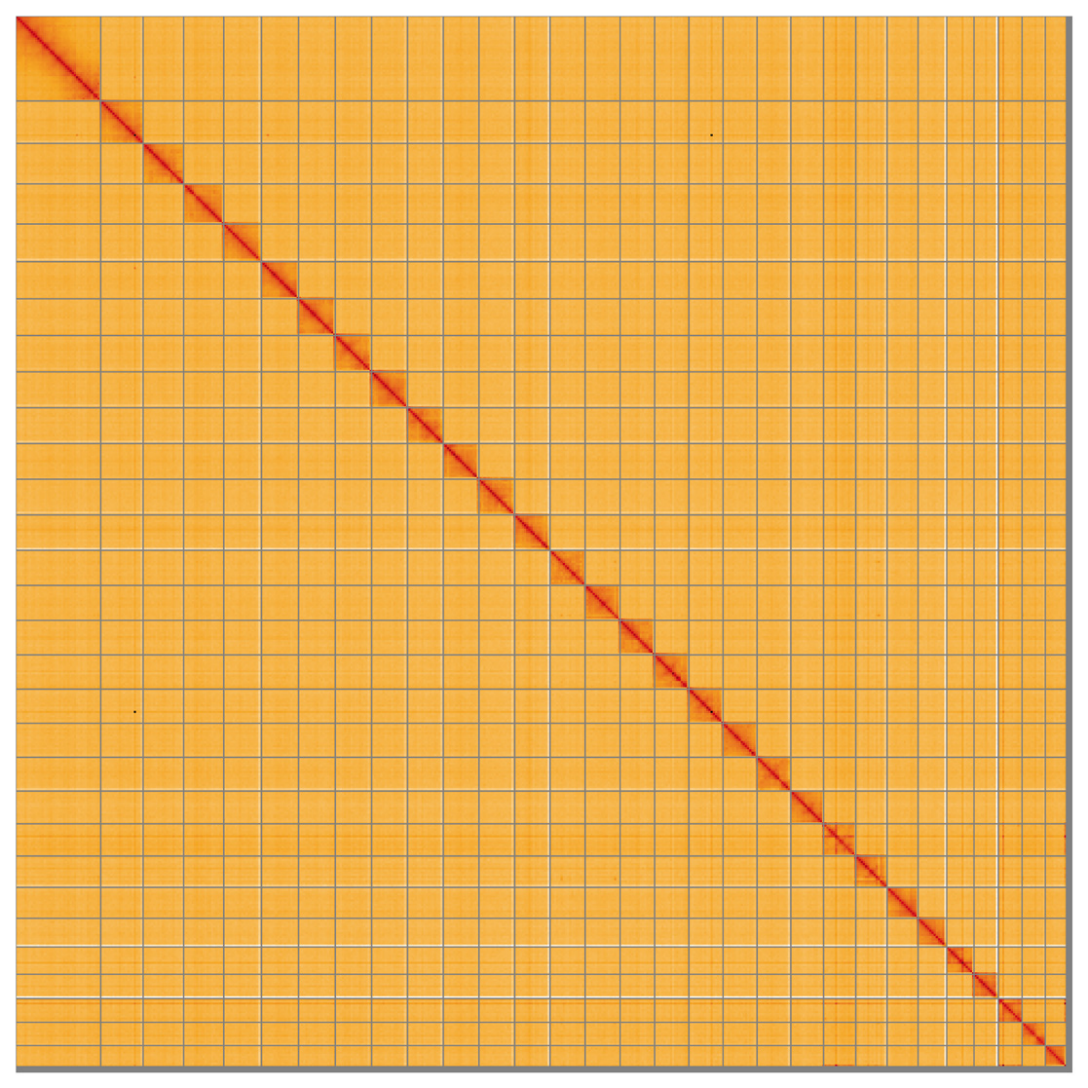
Genome assembly of *Polia nebulosa* ilPolNebu2.1: Hi-C contact map of the ilPolNebu2.1 assembly, visualised using HiGlass. Chromosomes are shown in order of size from left to right and top to bottom. An interactive version of this figure may be viewed at https://genome-note-higlass.tol.sanger.ac.uk/l/?d=Zi0bGGmlSG-ARGXFd85SkA.

Most of the assembly sequence (99.27%) was assigned to 30 chromosomal-level scaffolds, representing 29 autosomes and the Z sex chromosome. These chromosome-level scaffolds, confirmed by the Hi-C data, are named in order of size ( Figure 5; Table 3). During manual curation it was noted that Chromosome Z was assigned by synteny to *Mythimna impura* (GCA_905147345.3).

**Table 3.** Chromosomal pseudomolecules in the genome assembly of *Polia nebulosa*, ilPolNebu2.

INSDC accession	Name	Length (Mb)	GC%
OX589588.1	1	40.57	39.0
OX589589.1	2	38.69	39.0
OX589590.1	3	38.32	39.0
OX589591.1	4	36.01	39.0
OX589592.1	5	35.45	39.0
OX589593.1	6	35.16	39.0
OX589594.1	7	34.53	39.0
OX589595.1	8	34.3	39.0
OX589596.1	9	34.18	39.0
OX589597.1	10	34.17	39.0
OX589598.1	11	34.12	39.0
OX589599.1	12	33.7	39.0
OX589600.1	13	33.5	39.5
OX589601.1	14	33.46	39.0
OX589602.1	15	32.91	39.5
OX589603.1	16	32.84	38.5
OX589604.1	17	32.6	39.0
OX589605.1	18	32.5	39.5
OX589606.1	19	32.22	39.5
OX589607.1	20	31.35	39.0
OX589608.1	21	30.62	39.5
OX589609.1	22	30.01	39.0
OX589610.1	23	29.58	39.0
OX589611.1	24	27.4	39.0
OX589612.1	25	26.09	39.0
OX589613.1	26	23.15	39.0
OX589614.1	27	22.66	39.5
OX589615.1	28	22.28	40.0
OX589616.1	29	20.08	40.0
OX589587.1	Z	80.95	38.5
OX589617.1	MT	0.02	19.5

While not fully phased, the assembly deposited is of one haplotype. Contigs corresponding to the second haplotype have also been deposited. The mitochondrial genome was also assembled and can be found as a contig within the multifasta file of the genome submission, and as a separate fasta file.

The final assembly has a Quality Value (QV) of 63.7. The *k*-mer completeness of the primary assembly was estimated as 79.17%, alternate haplotype 76, 79%, and of the combined assemblies was 98.81%. BUSCO (v5.4.3) analysis using the lepidoptera_odb10 reference set ( *n* = 5,286) indicated a completeness score of 98.9% (single = 98.0%, duplicated = 0.9%).

## Methods

### Sample acquisition

The specimen of *Polia nebulosa* used for PacBio HiFi sequencing (specimen ID SAN00002623, ToLID ilPolNebu2) was collected from Glen Strathfarrar, Highlands, Scotland, United Kingdom (latitude 57.41, longitude –4.73) on 2022-06-27, using a moth trap. The specimen was collected by Andy Griffiths (Wellcome Sanger Institute) and identified by Tom Prescott (Butterfly Conservation) and preserved by flash freezing.

The specimen used for Hi-C sequencing (specimen ID SAN00002592, ToLID ilPolNebu1) was an adult specimen collected from Mount Stuart, Isle of Bute, Argyll and Bute, Scotland, United Kingdom (latitude 55.8, longitude –5.03) on 2022-07-02, using a moth trap. The specimen was collected and identified by Ronald Forrester (Bute Natural History Society) and preserved on dry ice.

### Nucleic acid extraction

The workflow for high molecular weight (HMW) DNA extraction at the Wellcome Sanger Institute (WSI) Tree of Life Core Laboratory includes a sequence of procedures: sample preparation and homogenisation, DNA extraction, fragmentation and purification. Detailed protocols are available on protocols.io ( Denton *et al.*, 2023b). The ilPolNebu2 sample was prepared for DNA extraction by weighing and dissecting it on dry ice ( Jay *et al.*, 2023). Tissue from the thorax was homogenised using a PowerMasher II tissue disruptor ( Denton *et al.*, 2023a).

HMW DNA was extracted in the WSI Scientific Operations core using the Automated MagAttract v2 protocol ( Oatley *et al.*, 2023). The DNA was sheared into an average fragment size of 12–20 kb in a Megaruptor 3 system ( Bates *et al.*, 2023). Sheared DNA was purified by solid-phase reversible immobilisation, using AMPure PB beads to eliminate shorter fragments and concentrate the DNA ( Strickland *et al.*, 2023). The concentration of the sheared and purified DNA was assessed using a Nanodrop spectrophotometer and Qubit Fluorometer using the Qubit dsDNA High Sensitivity Assay kit. Fragment size distribution was evaluated by running the sample on the FemtoPulse system.

RNA was extracted from thorax tissue of ilPolNebu2 in the Tree of Life Laboratory at the WSI using the RNA Extraction: Automated MagMax™ *mir*Vana protocol ( do Amaral *et al.*, 2023). The RNA concentration was assessed using a Nanodrop spectrophotometer and a Qubit Fluorometer using the Qubit RNA Broad-Range Assay kit. Analysis of the integrity of the RNA was done using the Agilent RNA 6000 Pico Kit and Eukaryotic Total RNA assay.

### Library preparation and sequencing

Libraries were prepared using the PacBio Express Template Preparation Kit v2.0 (Pacific Biosciences, California, USA) as per the manufacturer's instructions. The kit includes the reagents required for removal of single-strand overhangs, DNA damage repair, end repair/A-tailing, adapter ligation, and nuclease treatment. Library preparation also included a library purification step using AMPure PB beads (Pacific Biosciences, California, USA) and size selection step to remove templates <3kb using AMPure PB modified SPRI. DNA concentration was quantified using the Qubit Fluorometer v2.0 (Thermo Fisher Scientific) and Qubit HS Assay Kit and the final library fragment size analysis was carried out using the Agilent Femto Pulse Automated Pulsed Field CE Instrument (Agilent Technologies).

HMW DNA samples were sequenced using the Sequel IIe system (Pacific Biosciences, California, USA). The concentration of the library loaded onto the Sequel IIe was between 40 – 135 pM. The SMRT link software, a PacBio web-based end-to-end workflow manager, was used to set-up and monitor the run, as well as perform primary and secondary analysis of the data upon completion.

Hi-C data were generated from the head of the ilPolNebu1 using the Arima-HiC v2 kit. In brief, 20–50mg of frozen tissue (stored at –80°C) was fixed, and the DNA crosslinked using a TC buffer with 22% formaldehyde concentration. After crosslinking the tissue was homogenised using the Diagnocine Power Masher-II and BioMasher-II tubes and pestles. Following the Arima-HiC v2 kit manufacturer's instructions, crosslinked DNA was digested using a restriction enzyme master mix. The 5’-overhangs were filled in and labelled with biotinylated nucleotides and proximally ligated. An overnight incubation was carried out for enzymes to digest remaining proteins and for crosslinks to reverse. A clean up was performed with SPRIselect beads prior to library preparation. Additionally biotinylation percentage was estimated using the Qubit Fluorometer v2.0 (Thermo Fisher Scientific) and Qubit HS Assay Kit and Arima-HiC v2 QC beads.

For Hi-C library preparation, DNA was fragmented using the Covaris E220 sonicator (Covaris) and size selected using SPRISelect beads to 400 to 600 bp. The DNA was then enriched using the Arima-HiC v2 kit Enrichment beads. Using the NEBNext Ultra II DNA Library Prep Kit (New England Biolabs) for end repair, a-tailing, and adapter ligation. This uses a custom protocol which resembles the standard NEBNext Ultra II DNA Library Prep protocol but where library preparation occurs while DNA is bound to the Enrichment beads. For library amplification, 10–16 PCR cycles were required, determined by the sample biotinylation percentage. The Hi-C sequencing was performed using paired-end sequencing with a read length of 150 bp on an Illumina NovaSeq 6000.

Poly(A) RNA-Seq libraries were constructed using the NEB Ultra II RNA Library Prep kit, following the manufacturer’s instructions. RNA sequencing was performed on the Illumina NovaSeq 6000 instrument.

### Genome assembly, curation and evaluation

***Assembly***

The HiFi reads were first assembled using Hifiasm ( Cheng *et al.*, 2021) with the --primary option. Haplotypic duplications were identified and removed using purge_dups ( Guan *et al.*, 2020). The Hi-C reads were mapped to the primary contigs using bwa-mem2 ( Vasimuddin *et al.*, 2019). The contigs were further scaffolded using the provided Hi-C data ( Rao *et al.*, 2014) in YaHS ( Zhou *et al.*, 2023) using the --break option for handling potential misassemblies. The scaffolded assemblies were evaluated using Gfastats ( Formenti *et al.*, 2022), BUSCO ( Manni *et al.*, 2021) and MERQURY.FK ( Rhie *et al.*, 2020).

The mitochondrial genome was assembled using MitoHiFi ( Uliano-Silva *et al.*, 2023), which runs MitoFinder ( Allio *et al.*, 2020) and uses these annotations to select the final mitochondrial contig and to ensure the general quality of the sequence.

***Assembly curation***

The assembly was decontaminated using the Assembly Screen for Cobionts and Contaminants (ASCC) pipeline (article in preparation). Flat files and maps used in curation were generated in TreeVal ( Pointon *et al.*, 2023). Manual curation was primarily conducted using PretextView ( Harry, 2022), with additional insights provided by JBrowse2 ( Diesh *et al.*, 2023) and HiGlass ( Kerpedjiev *et al.*, 2018). Scaffolds were visually inspected and corrected as described by Howe *et al*. (2021). Any identified contamination, missed joins, and mis-joins were corrected, and duplicate sequences were tagged and removed. The sex chromosome was assigned by synteny analysis. The curation process is documented at https://gitlab.com/wtsi-grit/rapid-curation (article in preparation).

***Evaluation of the final assembly***

A Hi-C map for the final assembly was produced using bwa-mem2 ( Vasimuddin *et al.*, 2019) in the Cooler file format ( Abdennur & Mirny, 2020). To assess the assembly metrics, the *k*-mer completeness and QV consensus quality values were calculated in Merqury ( Rhie *et al.*, 2020). This work was done using Nextflow ( Di Tommaso *et al.*, 2017) DSL2 pipelines “sanger-tol/readmapping” ( Surana *et al.*, 2023a) and “sanger-tol/genomenote” ( Surana *et al.*, 2023b). The genome was analysed within the BlobToolKit environment ( Challis *et al.*, 2020) and BUSCO scores ( Manni *et al.*, 2021) were calculated.

The genome evaluation pipelines were developed using nf-core tooling ( Ewels *et al.*, 2020) and MultiQC ( Ewels *et al.*, 2016), relying on the Conda package manager, the Bioconda initiative ( Grüning *et al.*, 2018), the Biocontainers infrastructure ( da Veiga Leprevost *et al.*, 2017), as well as the Docker ( Merkel, 2014) and Singularity ( Kurtzer *et al.*, 2017) containerisation solutions.

Table 4 contains a list of relevant software tool versions and sources.

**Table 4.** Software tools: versions and sources.

Software tool	Version	Source
BEDTools	2.30.0	https://github.com/arq5x/bedtools2
BLAST	2.14.0	ftp://ftp.ncbi.nlm.nih.gov/blast/executables/blast+/
BlobToolKit	4.3.7	https://github.com/blobtoolkit/blobtoolkit
BUSCO	5.4.3 and 5.5.0	https://gitlab.com/ezlab/busco
bwa-mem2	2.2.1	https://github.com/bwa-mem2/bwa-mem2
Cooler	0.8.11	https://github.com/open2c/cooler
DIAMOND	2.1.8	https://github.com/bbuchfink/diamond
fasta_windows	0.2.4	https://github.com/tolkit/fasta_windows
FastK	427104ea91c78c3b8b8b49f1a7d6bbeaa869ba1c	https://github.com/thegenemyers/FASTK
Gfastats	1.3.6	https://github.com/vgl-hub/gfastats
GoaT CLI	0.2.5	https://github.com/genomehubs/goat-cli
Hifiasm	0.19.8-r587	https://github.com/chhylp123/hifiasm
HiGlass	44086069ee7d4d3f6f3f0012569789ec138f42b84 aa44357826c0b6753eb28de	https://github.com/higlass/higlass
Merqury.FK	d00d98157618f4e8d1a9190026b19b471055b22e	https://github.com/thegenemyers/MERQURY.FK
MitoHiFi	3	https://github.com/marcelauliano/MitoHiFi
MultiQC	1.14, 1.17, and 1.18	https://github.com/MultiQC/MultiQC
NCBI Datasets	15.12.0	https://github.com/ncbi/datasets
Nextflow	23.04.0-5857	https://github.com/nextflow-io/nextflow
PretextView	0.2.5	https://github.com/sanger-tol/PretextView
purge_dups	1.2.5	https://github.com/dfguan/purge_dups
samtools	1.16.1, 1.17, and 1.18	https://github.com/samtools/samtools
sanger-tol/ascc	-	https://github.com/sanger-tol/ascc
sanger-tol/ genomenote	1.1.1	https://github.com/sanger-tol/genomenote
sanger-tol/ readmapping	1.2.1	https://github.com/sanger-tol/readmapping
Seqtk	1.3	https://github.com/lh3/seqtk
Singularity	3.9.0	https://github.com/sylabs/singularity
TreeVal	1.0.0	https://github.com/sanger-tol/treeval
YaHS	1.2a.2	https://github.com/c-zhou/yahs

### Wellcome Sanger Institute – Legal and Governance

The materials that have contributed to this genome note have been supplied by a Darwin Tree of Life Partner. The submission of materials by a Darwin Tree of Life Partner is subject to the **‘Darwin Tree of Life Project Sampling Code of Practice’**, which can be found in full on the Darwin Tree of Life website here. By agreeing with and signing up to the Sampling Code of Practice, the Darwin Tree of Life Partner agrees they will meet the legal and ethical requirements and standards set out within this document in respect of all samples acquired for, and supplied to, the Darwin Tree of Life Project.

Further, the Wellcome Sanger Institute employs a process whereby due diligence is carried out proportionate to the nature of the materials themselves, and the circumstances under which they have been/are to be collected and provided for use. The purpose of this is to address and mitigate any potential legal and/or ethical implications of receipt and use of the materials as part of the research project, and to ensure that in doing so we align with best practice wherever possible. The overarching areas of consideration are:

•   Ethical review of provenance and sourcing of the material

•   Legality of collection, transfer and use (national and international)

Each transfer of samples is further undertaken according to a Research Collaboration Agreement or Material Transfer Agreement entered into by the Darwin Tree of Life Partner, Genome Research Limited (operating as the Wellcome Sanger Institute), and in some circumstances other Darwin Tree of Life collaborators.

## Data Availability

European Nucleotide Archive: Polia nebulosa (grey arches). Accession number PRJEB61359; https://identifiers.org/ena.embl/PRJEB61359. The genome sequence is released openly for reuse. The *Polia nebulosa* genome sequencing initiative is part of the Darwin Tree of Life (DToL) project. All raw sequence data and the assembly have been deposited in INSDC databases. The genome will be annotated using available RNA-Seq data and presented through the Ensembl pipeline at the European Bioinformatics Institute. Raw data and assembly accession identifiers are reported in Table 1 and Table 2.

## Author information

Members of the Wellcome Sanger Institute Tree of Life Management, Samples and Laboratory team are listed here: https://doi.org/10.5281/zenodo.12162482.

Members of Wellcome Sanger Institute Scientific Operations: Sequencing Operations are listed here: https://doi.org/10.5281/zenodo.12165051.

Members of the Wellcome Sanger Institute Tree of Life Core Informatics team are listed here: https://doi.org/10.5281/zenodo.12160324.

Members of the Tree of Life Core Informatics collective are listed here: https://doi.org/10.5281/zenodo.12205391.

Members of the Darwin Tree of Life Consortium are listed here: https://doi.org/10.5281/zenodo.4783558.
