# Supplementary material for: The genome sequence of the Grey Arches moth, Polia nebulosa Hufnagel, 1766
Source: Wellcome Open Res. 2025 Feb 3;10:39. [Version 1] doi: 10.12688/wellcomeopenres.23608.1 (PMC11971629; doi:10.12688/wellcomeopenres.23608.1)
Supplement: Peer review history

# Reviewer report: Reviewer response for version 1

**DOI:** 10.21956/wellcomeopenres.26044.r120774

**Published:** 2025-04-04

**Authors:** Željko Tomanović

**Affiliations:** 1 Serbia University of Belgrade, Belgrade, Serbia

The authors presented the genome assembly of a single male specimen of the moth *Polia nebulosa Hufnagel*, 1766 , commonly known as the Grey Arches. In the background, the authors gave nice and appropriate information about the biology and distribution of the species. They also compiled the complete mitochondrial genome. The authors presented a genome assembly with a total length of 1,011.20 Mb in 114 sequence scaffolds represented by 29 autosomes and sex chromosome Z. I recommend paper for indexing.

Are sufficient details of methods and materials provided to allow replication by others?

Yes

Is the rationale for creating the dataset(s) clearly described?

Yes

Are the datasets clearly presented in a useable and accessible format?

Yes

Are the protocols appropriate and is the work technically sound?

Yes

Reviewer Expertise:

biodiversity, phylogenetics, taxonomy

I confirm that I have read this submission and believe that I have an appropriate level of expertise to confirm that it is of an acceptable scientific standard.

# Reviewer report: Reviewer response for version 1

**DOI:** 10.21956/wellcomeopenres.26044.r118343

**Published:** 2025-02-15

**Authors:** Sivasankaran Kuppusamy

**Affiliations:** 1 Loyola College, Chennai, Tamil Nadu, India

The authors have done chromosome-level genome assembly of *Polia nebulosa *Hufnagel, 1766. They assembled and produced a total length of 1,011.20 Mb genome sequence. During the curation, authors have observed a synteny chromosome. The work was commendable and robust data is useful for the phylogenomic analysis. 

 In the background first sentence

 The species *Polia nebulosa *was first described by Hufnagel in 1766. Authors have mentioned Hufnagel’s surname i.e., Johann Siegfried Hufnagel 1766. No need to mention the surname here. Surname can be deleted.

 Throughout the article, the authors have used the full name of the genus, *Polia nebulosa*. First time, the genus name may be given in full form; later, it may be shortened to *P. nebulosa.*

 Here we present a chromosomally complete genome sequence for *P. nebulosa. *The sentence can be modified as “Here we present a chromosome-level complete genome assembly of *P. nebulosa…….”*

 Authors have assembled the sequence of 1,011.20 megabase in size. In the comprehensive data authors haven’t detected protein-coding genes and gene transcripts. What was the reason?

 Above all, I confirm that the manuscript meets the necessary scientific standard and is suitable for indexing.

Are sufficient details of methods and materials provided to allow replication by others?

Yes

Is the rationale for creating the dataset(s) clearly described?

Yes

Are the datasets clearly presented in a useable and accessible format?

Yes

Are the protocols appropriate and is the work technically sound?

Yes

Reviewer Expertise:

Phylogeny of Lepidopteran moths

I confirm that I have read this submission and believe that I have an appropriate level of expertise to confirm that it is of an acceptable scientific standard.
